# Low-Cost Optical pH Sensor with a Polyaniline (PANI)-Sensitive Layer Based on Commercial Off-the-Shelf (COTS) Components

**DOI:** 10.3390/mi14122197

**Published:** 2023-11-30

**Authors:** Serguei Stoukatch, Marc Debliquy, Francois Dupont, Jean-Michel Redouté

**Affiliations:** 1 Microsys Laboratory, Department of Electrical Engineering and Computer Science (Montefiore Institute), University of Liège, 4000 Liège, Belgium; fff.dupont@uliege.be (F.D.); jean-michel.redoute@uliege.be (J.-M.R.); 2 Service de Science des Matériaux, Faculté Polytechnique, Université de Mons, 7000 Mons, Belgium; marc.debliquy@umons.ac.be

**Keywords:** optical pH sensor, colorimetric characterization, optical sensor, polyaniline, COTS components

## Abstract

In this paper, we presented a novel, compact, conceptually simple, and fully functional low-cost prototype of a pH sensor with a PANI thin film as a sensing layer. The PANI deposition process is truly low-cost; it performs from the liquid phase, does not required any specialized equipment, and comprises few processing steps. The resulting PANI layer has excellent stability, resistance to solvents, and bio- and chemical compatibility. The pH sensor’s sensing part includes only a few components such as a red-light-emitting diode (LED) as a light source, and a corresponding photodiode (PD) as a detector. Unlike other PANI-based sensors, it requires no sophisticated and expensive techniques and components such lasers to excite the PANI or spectrometry to identify the PANI color change induced by pH variation. The pH sensor is sensitive in the broad pH range of 3 to 9, which is useful for numerous practical applications. The sensor requires a tiny volume of the test specimen, as little as 55 µL. We developed a fully integrated packaging solution for the pH sensor that comprises a limited number of components. The pH sensor comprises exclusively commercial off-the-shelf (COTS) components and standard printed circuit boards. The pH sensor is assembled using standard surface mounting technology (SMT).

## 1. Introduction

There are various methods of measuring pH which can be divided in two main groups [1]: the electro-metric or -chemical methods, which are based on using electrodes for measuring a voltage change, and the colorimetric methods, which are using pH-sensitive color-changing indicators, typically in the form of liquid solutions, papers, or indicators immobilized in a porous matrix such as, e.g., a polymer or a sol-gel. The electro-metric or -chemical methods [2] are based on measuring the potential difference between two electrodes, a measuring and a reference electrode, which are dipped into the liquid specimen. The potential difference between those electrodes is a function of the hydrogen ion activity and defines the pH of the specimen [3]. Based on experts’ recommendations [2,4] such methods are set by ISO [5] as the preferred method for determining the pH value in aqueous solution. The standard also defines the instrumentation and the required buffer solutions for calibrating the instrument.

The electrochemical methods are indeed the most accurate, repeatable, and well established. However, often there are needs for an alternative technique, aiming for quicker results, and requiring a low-cost, less complex, and more portable instrumentation. Moreover, the electrochemical methods require frequent calibration. The numerous colorimetric methods are a valuable alternative for that. 

Colorimetry is a particular case of commonly used optical detection methods, which are widely used for fluid analyses in chemical and bio-medical applications [6]. The intensity of the color depends on the concentration of the analyte in the liquid, and the results can be interpreted either directly, without any equipment, or using a dedicated equipment if a high-accuracy measurement is required. 

The best-known low cost and rapid colorimetric method for pH determination is the paper-based universal indicator that consists of a paper strip impregnated with a mixture of pH-sensitive color-changing compounds, resulting in a continuous color change in the paper according to the pH of the analyte it is dipped into [3]. The color change can be quantified by human eye or by optical instruments, including spectrometers or relatively simpler optical sensing systems, such as photodiodes or cameras. In paper [7], the authors reported that a smartphone-based spectrometer is used for dye-assisted pH detection in the range of 6–8 pH unit. 

In line with the cost-reduction trend, research efforts are increasing to find alternatives to expensive spectrometers, leading researchers to look for a simple optical sensor system. A good example of the successful replacement of an expensive spectrometer with a light emitting diode (LED)-based colorimetric system is reported in [8].

Some companies have already successfully commercialized optical pH sensors. For example, Hamilton Company [9] offers a sensing element in the form of spot or patch that is composed of a fluorescent dye embedded in a hydrophilic gel compound. The product has fundamental limitation on the pH measurement range, which is limited within the range of 5 to 9 due to the luminescent properties of specifical sensing material.

Pyroscience [10] sells two types of optical sensors: fixed fiber pH mini-sensor and pH sensors spot. They are available in different configurations but have a limited measurement range. In addition to the measurement range, the most common limitation is the response time, which is typically between 30 s and 120 s. A notable point of concern for colorimetric methods is cross-sensitivity to certain chemicals. Some chemicals, especially organic solvents, can attack the sensitive layer, causing it to lose its measuring ability, or in extreme cases, damage it irreversibly. 

Among colorimetric methods, a remarkable place is taken by pH sensors that use a polyaniline (PANI) as the sensitive material to pH variation [11]. PANI is a well-studied conductive polymer [12] that presents a pH-dependent absorption coefficient in the visible light and partially in the near-infrared region (NIR) of the 0.4–1 µm range [13]. PANI has several other properties that make it interesting to use as a material for pH sensors for chemical and biomedical applications [12]. The most important ones are excellent stability, resistance to solvents, and bio- and chemical compatibility [14]. In [13], the authors analyzed the broad NIR spectrum of PANI to detect the pH of the analyte. The article [15] reported on the use of a localized surface plasmon resonance (LSPR) to analyze a PANI–gold nanostructures as an optical sensor for monitoring the pH of saliva. In [16], the authors developed a PANI-tilted fiber Bragg grating (FBG) sensing head that also needs a FBG interrogator. 

In this paper, we present a low-cost prototype of a pH sensor with a PANI thin film as a sensing layer. The color change in the PANI layer is monitored using an LED and a photodiode (PD). Unlike other PANI-based sensors, it requires no sophisticated techniques such as lasers to excite PANI or spectrometry to identify the PANI color change. 

This paper is structured as follows. In the second section, we introduce the working principle of the optical pH sensor, and describe the concept and its architecture. In Section 2, we present the assembly technique and process flow applied for manufacturing the pH sensor. It also lists the materials used for the manufacturing of the pH sensor. In Section 3, we report and discuss the results of optical characterization. In the same section, we performed pH measurements in the test liquid specimen. In Section 4, we discussed the obtained results. Finally, we draw conclusions in Section 5.

## 2. Materials and Methods

### 2.1. Concept and Architecture of the Optical pH Sensor

The working principle of the optical pH sensor is straightforward. The sensitive PANI layer is dipped into the liquid specimen, whose pH must be determined, leading to a corresponding color change. The PANI turns its appearance from green in an acidic medium to blue purple in an alkaline medium. This color change is subsequently quantified by the LED and PD setup. 

**Figure 1:**
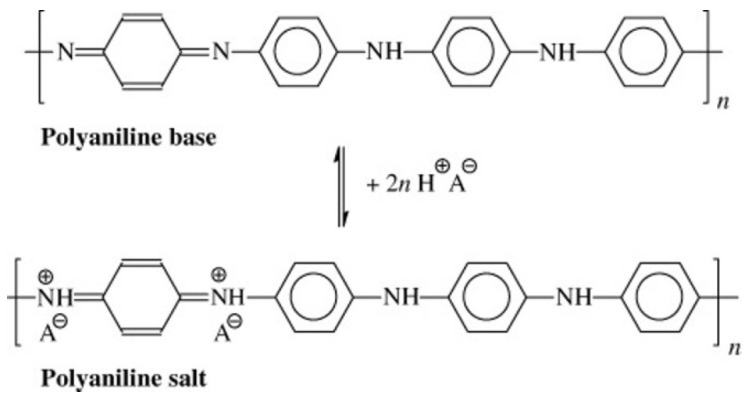
PANI (emeraldine) base reacts with an acid (HA) to yield the PANI (emeraldine) salt. A− is an arbitrary anion. Copyright © 2023 Society of Chemical Industry. Reprinted from [17] with permission from John Wiley and Sons.

PANI in the presence of acidic or basic media, in the pH range of 2 to 10, has the ability to reversibly undergo protonation (green form) and deprotonation (blue form) from its emeraldine base form to the emeraldine salt form and back. The mechanism of the protonation is illustrated in Figure 1.

**Figure 2:**
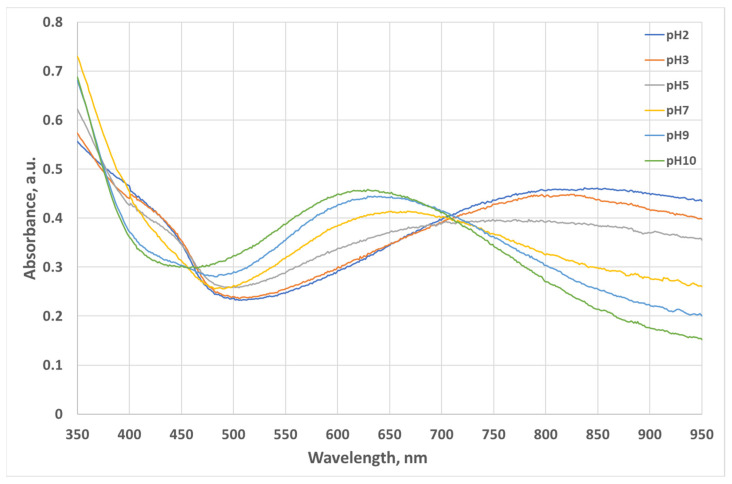
Absorption spectrum of an 8 µm thick PANI film deposited on a glass substrate versus pH (from pH2 to pH10). pH2 corresponds to the green form of PANI and pH10 corresponds to its blue form.

The two forms have different optical absorption spectrums. The absorption spectrum versus pH (pH2 to pH10) of an 8 µm thick PANI film deposited on a glass substrate is shown in Figure 2. It was measured by a spectrophotometer model UV/Vis lambda 365 from Perkin Elmer Inc., (Waltham, MA, USA) [18]. Perkin Elmer UV/Vis Lambda 365 Spectrophotometer is a precise laboratory table-based system that measures spectra in an operating range of 190 nm to 1100 nm. 

**Figure 3:**
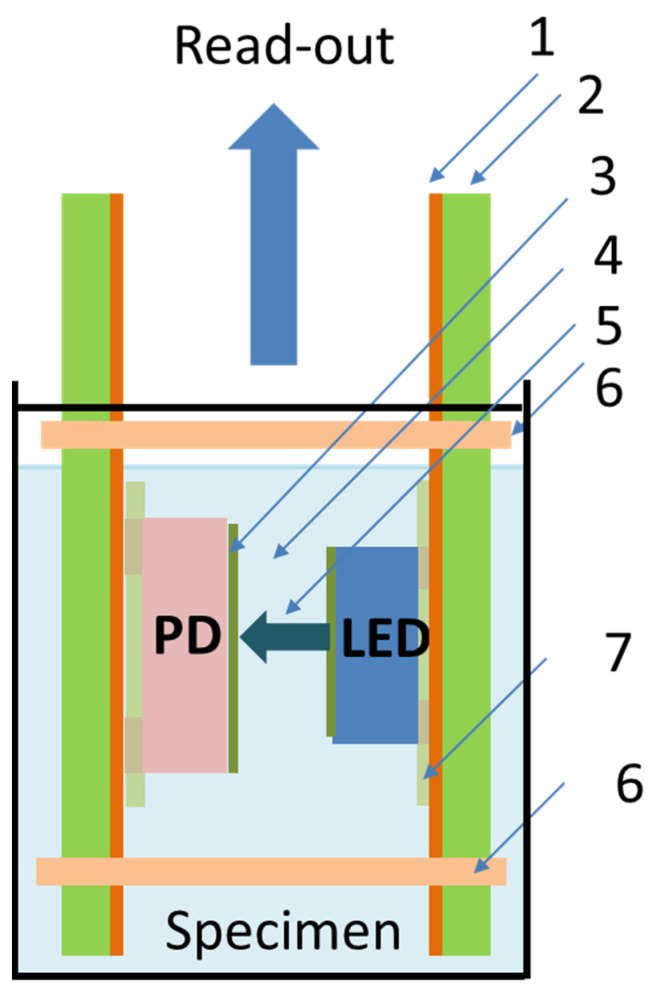
Concept of the optical pH sensor based on a PANI sensing layer: (1) the solder mask, (2) the thin PCB, (3) the sensing layer, (4) the sensing gap, (5) the light emitted by the LED, (6) the guide (optional features to vary the sensing gap), (7) the underfill layer.

The working principles and concept of the optical pH sensor are illustrated in Figure 3.

**Figure 4:**
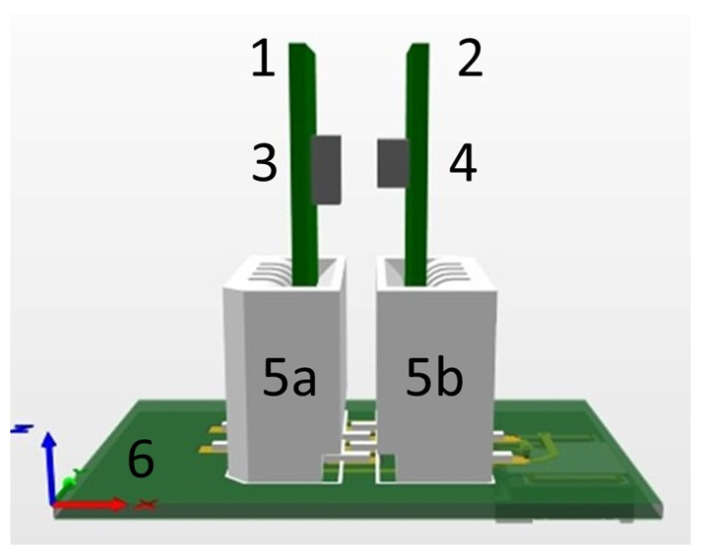
3D drawings of the optical pH sensor based on polyaniline: (1) PD PCB, (2) LED PCB, (3) PD, (4) LED, (5a,b), PD and LED connectors, (6) main PCB.

To go from concept to practical implementation, we need to design a packaging solution for the pH sensor. Unlike integrated circuit (IC) packaging, there is no uniform packaging solution for a wide range of sensors [19], including pH sensors. The sensors’ specificity dictates a specific requirement for the corresponding package. Additionally, for general requirements, the package for the pH sensor must withstand direct contact with a liquid specimen. Based on that, we developed a fully integrated packaging solution for the pH sensor that comprises a limited number of components: a LED and a PD which are mounted on custom-designed PCBs. The LED and PD PCBs are connected electrically to the mother board by means of two standard connectors. The connectors are “easy-on”, 1 mm pitch 4 way straight female connector with a vertical contact, from manufacturer Molex [20], as presented in Figure 4.

The 8 µm thick PANI thin layer was deposited on a polyethylene terephthalate (PET) thin film (25 µm thickness) using the process developed and reported in [21]. The choice of PET as a material for the PANI carrier was due to its unique physical and chemical properties in combination with low cost and easy availability. Indeed, PET has a low moisture absorption of 0.05–0.1%, adequate mechanical strength, and good thermal stability [22]. Remarkably, it has a high (above 80%) [22] and almost constant optical transparency in the wavelength range of 320 to 1000 nm [23]. This makes PET a relevant candidate for this particular application. Then, the PANI-coated thin film was mounted between the LED and sensing surface of the PD. 

As the PANI layer displays pH-dependent absorption in the wavelength range between 400 and 1000 nm, the most straightforward way to perform pH measurement [24] is to illuminate it, when dipped into the analyte solution, with light from a broadband optical source and then to analyze the optical response with a standard spectrometer. 

In a laboratory environment, it can be an excellent match between a broadband optical source Model LS-1, from Ocean Optics [25], which provides a wavelength range of 350–1050 nm, and a standard spectrometer [26], for instance, a device from the Shimadzu [27] family with a wavelength range of 190–1100 nm. Since most spectrometers are bulky laboratory stationary devices, there are only a few sources of so-called compact and low-cost mini-spectrometers, often called polychromators. For example, Hamamatsu, Japan [28], supplies mini-spectrometers covering a spectral rang from ultraviolet (UV) to NIR. To cover the entire spectrum for PANI, it is necessary to use two mini-spectrometers C10988MA-01 and C11708MA with spectral response ranges of 340 to 750 nm and 640 to 1050 nm, respectively. The dimensional outline of the packaged component is 27.6 × 16.8 × 13 mm^3^ and weighs 9 g. The component has 10 terminals for mounting on a PCB. The manufacturer recommends soldering by hand or in a solder bath but warns that the component can be easily damaged by soldering. 

We explored an alternative solution for that, which has a lower cost and is more compact. In Figure 2, the entire PANI spectrum has at least two remarkable regions in which the difference in absorption of the PANI forms (from pH2 to pH10) is the most distinguishable. Those two regions are around 620–630 nm and 900–1000 nm. Instead of illuminating the PANI by a broadband light source, we considered illuminating it in one of these two regions. We selected the 620–630 nm region for the following reasons. First, red-light LEDs are easily available compared to NIR LEDs. Second, compact (in SMD format) NIR LEDs have lower radiometric power, typically in the range of 15–25 mW, versus red LED, typically of 80 mW and above [29]. Finally, an additional advantage to using a standard red LED instead of an NIR LED is that the red LED emits light in the visible spectrum, whereas an NIR LED emits light at above 950 nm, which is beyond the 700 nm wavelength of the upper range of the visible spectrum. We believe that it is an important advantage for using a device in the field environment. 

Therefore, we selected LUXEON Rubix 1416-L1RX-RED1000000000CT-ND red LED from Lumileds Holding B.V. [30] to illuminate the sensitive layer. This LED is in a surface mount device (SMD) format package. It has a peak wavelength between 620 and 630 nm. According to the technical data sheet [30], the LEDs are tested and binned with a constant drive current of 1500 mA (DC) at a junction temperature of 85 °C. Under these conditions, the LED delivers a typical radiometric power of 85 mW. 

To match the wavelength emitted by the LED, we selected a corresponding PD with a spectral bandwidth of 350 to 1120 nm, and a peak wavelength of 900 nm. The PD is TEMD7000X01 from Vishay Semiconductors [31]. It is also, like the LED, in an SMD package format. 

The selected LED and PD are compact components with a volume of 1.97 mm^3^ and 2.13 mm^3^, respectively, which is negligible compared to 6028 mm^3^ for the Hamamatsu mini-spectrometers. The LED and PD are SMD-format components that can be mounted on the PCB with a standard solder reflow process. 

All the components are commercial off-the-shelf (COTS) that are easily available and interchangeable. All 3 PCBs, namely the LED PCB, the FD PCB, and the motherboard PCBs, are standard and share the following common features, such as a low Tg FR4 with a standard epoxy-based solder mask, which is a lead-free solder reflow compliant. The PCBs are 0.5 mm thick, have a 0.7 mm diameter vias, and an 18 µm thick Cu track finished with an electroplated Ni/Au layer. All selected components are suitable for assembly using standard surface mounting technology (SMT). 

### 2.2. Assembly of the pH Sensor

For the assembly of the pH sensor, we used a standard assembly technique. The lead-free solder paste S3X70-M500D, from Koki, Japan [32], was applied on the corresponding PCB at room temperature by an automatic dispenser Nordson ASYMPTEK Spectrum S-820 [33] using an Archimedes spiral screw dispensing head. After that, we mounted SMD components such the LED, the PD, and the connector on the corresponding PCB using an automatic Autotronik SMT Pick & Place BS384V1 machine [34]. After that, the PCB was subjected to solder reflow using a PC-controlled reflow oven LPKF Protoflow S [35]. The reflow profile was in accordance with SMD components vendor’s recommendations [30,31] and compliant to reflow soldering guidelines (JEDEC J-STD-020D [36]). After the lead-free solder reflow, all LEDs and PDs were underfilled using an underfill (UF) ECCOBOND E 1172 A, from LOCTITE [37]. The UF is a low-viscosity epoxy-based material that fills in the gap between the assembly and PCB. 

**Figure 5:**
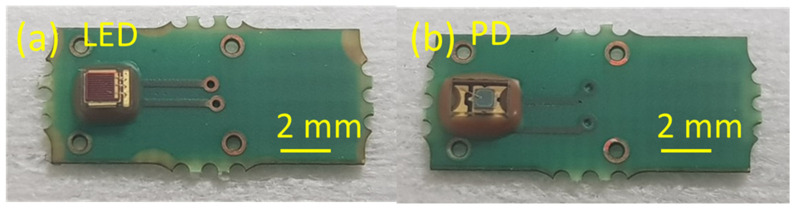
Fully assembled (mounted and underfilled) SMD on the corresponding PCBs: (**a**) LED and (**b**) PD.

**Figure 6:**
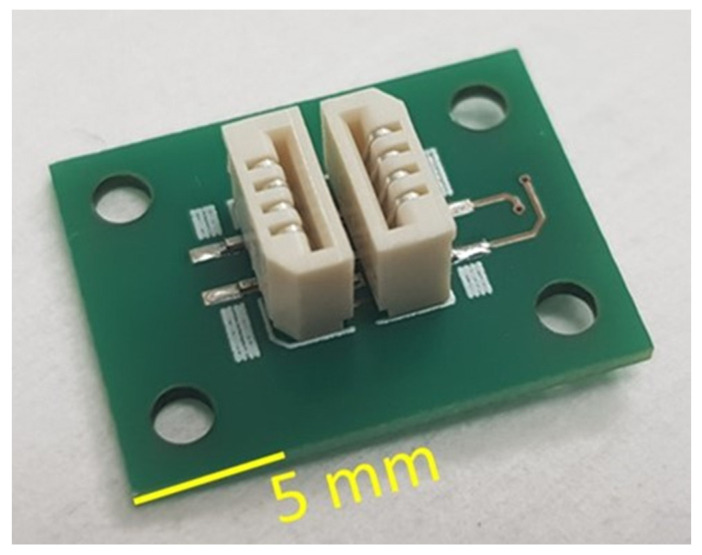
Two connectors mounted on the motherboard PCB.

The fully assembled SMD on the PCB, namely LED and PD, mounted and underfilled, are presented in Figure 5, and the mounted connector is presented in Figure 6.

On the LED and PD PCBs, we micromachined 4 through-holes that are foreseen to accommodate the 4 corresponding guides. The guides can align the LED and PD PCB according to each other and provide the possibility of adjusting the distance between the two PCBs. The distance between the LED and PD PCBs will define the gap between the LED and PD. Although such an option was not reported in the current publication, we used the connector (Figure 6) to hold the LED and PD PCBs.

The fully cured UF entirely fills in the gap between the SMD components (LED and PD) and the corresponding PCB and forms a fillet around each individual SMD components 0.5 mm wide and 0.5 mm high. The UF prevents a liquid test specimen from penetrating inside the gap between the SMD component and PCB, and covers electrical terminals on the PCB. Any possible contact between the electrical terminals and conductive tracks on the SMD components and PCB with the liquid specimen can potentially cause electrical shorts. The selected UF is a good electrical insulator with a volume resistivity of more than 10^15^ Ohm·m [37], has a relatively low moisture absorption (less than 1.5% [37]), and acts in the final assembly as an electrically insulating medium. Additionally, the UF also prevents any bare metal conductive tracks and terminals from becoming exposed to the liquid specimen and protects it from possible corrosion and other unwanted effects related to contact with the liquid specimen. 

**Figure 7:**
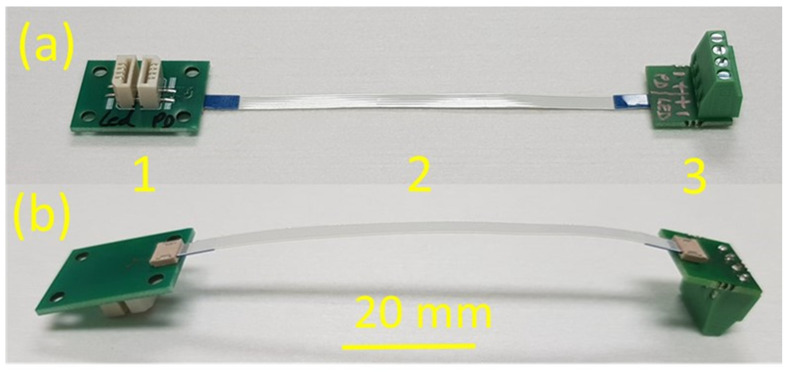
Photographs of a fully assembled sensing system: (**a**) face side angle view, (**b**) backside angle view. Where: (1) Connector PCB. (2) Cable. (3) Read-out PCB.

The motherboard PCB is electrically interconnected through an external cable to an interface PCB in order to ease the connection to the read-out system, as presented in Figure 7.

The cable can be easily manually inserted in and eventually retracted out the corresponding slots on the PCBs. 

**Figure 8:**
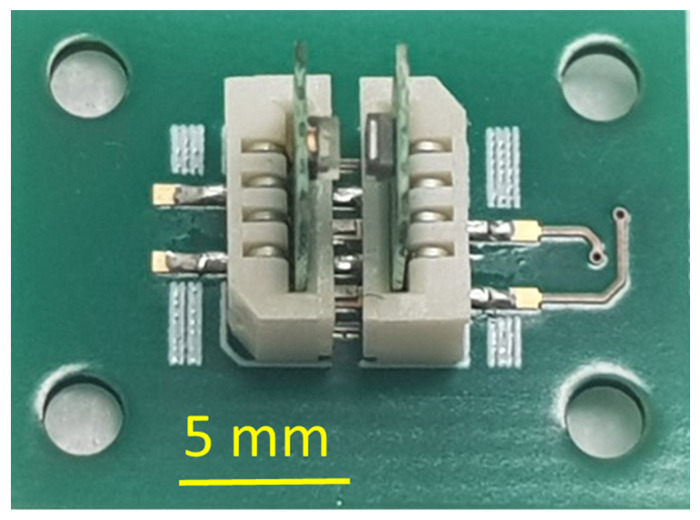
PCB with LED and PCB with PD mounted, inserted inside the two connectors.

The connector is designed in such a way that the LED and PD PCBs can be easily manually inserted inside the connector. If necessary, they can be also manually retracted from the slot. The connectors provide mechanical fixation and positions LED and PD PCB according to each other. The distance between the face side of LED and PD is fixed and equal to 0.8 mm. The connectors also provide an electrical interconnection between the connector PCB, and the LED and PD PCBs. Figure 8 illustrates the connector PCB with LED and PD PCB inserted. 

A folded plastic sheet made of PET covered on one face by PANI is inserted between the LED and PD. The 8 µm thick PANI layer is deposited directly onto the PET surface according to the procedure [21] by dipping the PET foils in a bath where the polymerization of PANI takes place. The process flow of PANI layer deposition is as follows. Initially, the PET foils were cleaned in a piranha mixture (mixture of 30% H2O2 and 96% H_2_SO_4_ in proportion 4:1), then rinsed with deionized water. After that, the PET foils are dipped in the bath. The 50 mL bath contains 1M hydrochloric acid (HCl, 541 µL HCl 37%), 457 µL aniline (linear molecular formula C_6_H_5_NH_2_), and 1141 g ammonium persulfate ((NH_4_)_2_SO_4_), which act as an oxidant. The temperature of the bath was kept constant at 30 °C under stirring. The dipping time was 10 min. Finally, the PET foil with the deposited 8 µm thick PANI layer was gently rinsed by dipping into a beaker with the deionized water. The properties of the PANI layers on PET foil were previously extensively characterized [38].

**Figure 9:**
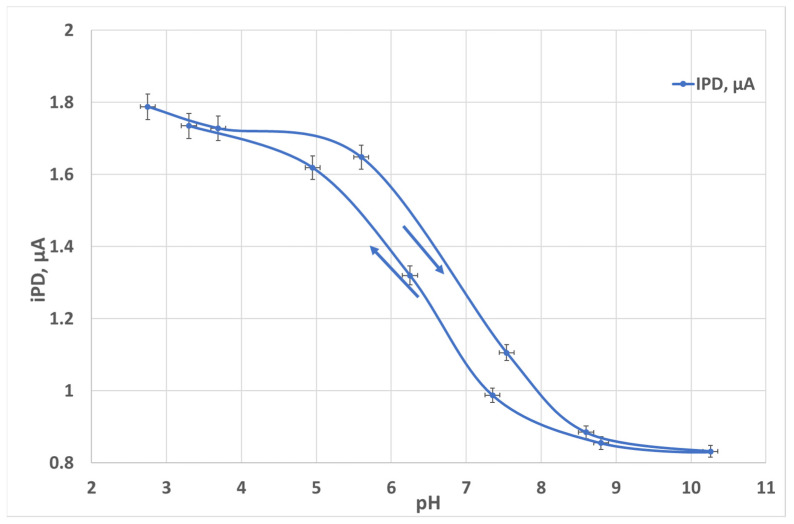
PD current (IPD) versus pH in the test solution.

The 8 µm thickness of the PANI layer was selected as a compromise between the signal intensity, the signal response and recovery [39,40] time, and the hysteresis behavior [41] of the PANI. The signal intensity is related to the absorbance (*A*) described by the Beer-Lambert law [42] that determines the concentration of chemical species that absorb light and expressed in Equation (1):

(1) $$A=\varepsilon \left(\lambda \right)\;l\;c\;$$

where *ε (λ)* is the molar absorptivity coefficient versus the wavelength, *l* is the optical path length, and *c* is the concentration. When the thicker layer (larger *l*) is higher, the absorbance of the PANI layer is higher and the signal intensity is stronger. Meanwhile, the response time and the recovery time are limited by the diffusion through the layer, which is roughly proportional to *l^2^*. The excessive PANI thickness may result in hysteresis behavior of the PANI layer. We extended our previous [21] work and we experimentally examined PANI thickness from 2 to 25 µm thick. The PANI films of 2 and 5 µm thickness did not exhibit a sufficient absorbance, and the films of 17 and 25 µm thickness demonstrated a significant measurement error due to the hysteresis phenomena as illustrated in Figure 9 for a 25 µm thick PANI layer. 

## 3. Results

### 3.1. Optics Characterization

**Figure 10:**
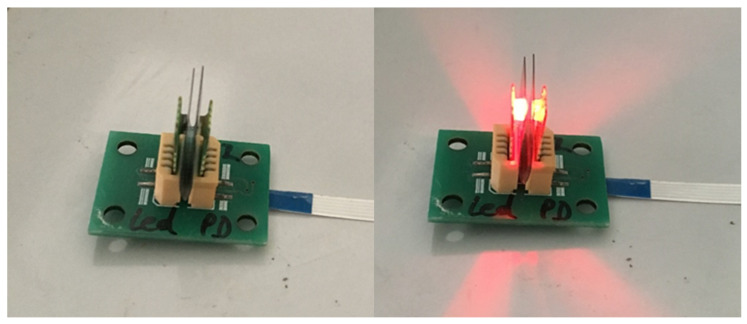
Linearity test, LED is off (**left**), LED is on (**right**).

As the first step of the sensor evaluation, we measured the PD response versus current in the LED. The purpose of the test was to evaluate the signal linearity. We also evaluated the impact of the ambient light. The measurements were performed in ambient air to be as close to the real application as possible. The test set up is presented in Figure 10.

**Figure 11:**
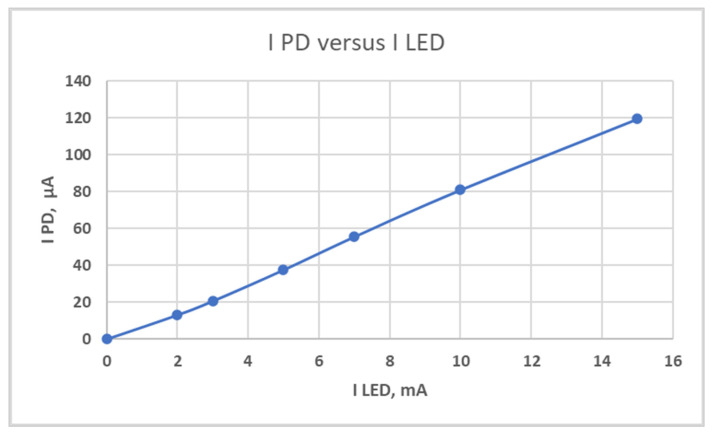
The PD current (I_PD_) as a function of the LED current (I_LED_), LED voltage is constant and equal to 1.7 V.

The voltage applied to the LED (threshold 1.7 V) is swept using a standard stabilized regulated power supply (Elix 20239) to vary the current in the LED. The PD current and the LED current are measured with a Keithley amperemeter (Keithley 6512) as a function of the LED current, as plotted in Figure 11. 

In the measured current range of 0 to 16 mA, the signal is quite linear. Although the sensor was not protected from an ambient natural light, and the PD current is negligibly low, at 0.085 µA when the LED is off.

### 3.2. pH Measurements

**Figure 12:**
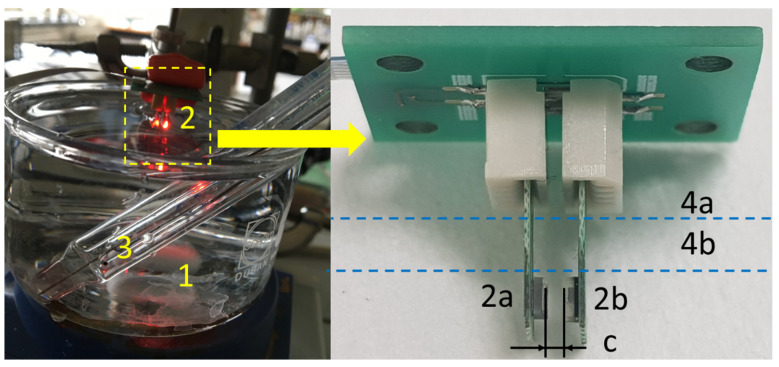
pH measurement setup, where (1) is a test specimen, (2) is a pH sensor, a PD (2a), and an LED (2b), (3) is a reference pH meter, Metrohm, and (4) is a level of the test specimen, where (4a) is the minimum level and (4b) is the maximum level of the test specimen, and (c) is a fixed measurement gap of 0.8 mm.

Once we performed the optical system characterization, the sensing system was ready for pH measurements. For that, the sensing system was directly immersed in a laboratory beaker with the test specimen. Specifically, only part of the sensing system comprising the LED, PD, and the PANI films mounted between them was dipped in the test specimen, as illustrated in Figure 12. 

**Figure 13:**
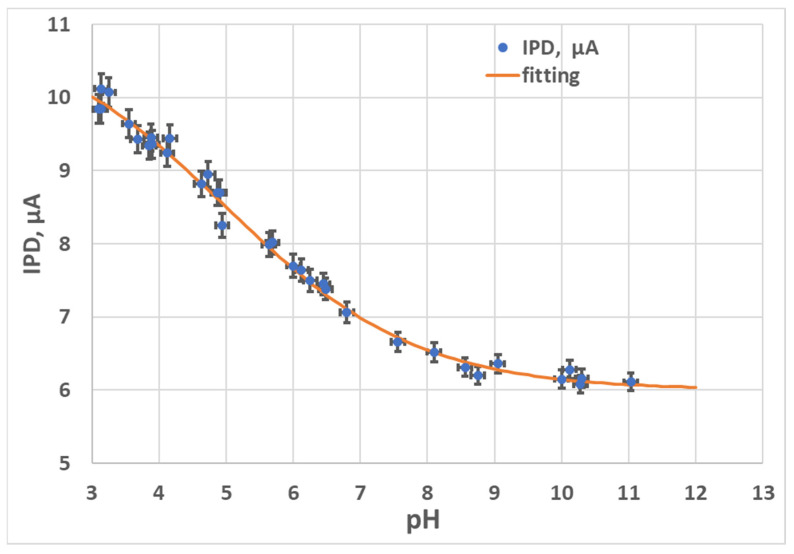
PD current (*I_PD_*) versus *pH* in the test solution.

The test specimen we selected was easily available 1 M NaCl aqueous solution. The pH of the test solution was adjusted in the pH range from 3 to 12 by incrementally adding 1 M HCl or 1M NaOH to the test solution to make it more acidic or more alkaline, respectively. The pH was constantly monitored by an external conventional reference pH meter with glass electrodes, from Metrohm [43], Switzerland. The PD response to various pH levels with a 1 min interval between each measurement point is plotted in the graph in Figure 13.

The orange curve corresponds to a sigmoid fitting of the experimental results. The PD current (*I_PD_*) is expressed in Equation (2).

(2) $$I_{PD}=6+\frac{5.05}{1+\exp\left(0.7\left(pH-5.1\right)\right)}$$

The results show a satisfactory response to pH with a sigmoidal shape without obvious hysteresis. 

## 4. Discussion

The response of the PD to the LED current is quite linear in a wide current range from 0 to 16 mA, which indicates a good match between the PD and the LED and the correct geometry of the entire optical system. The PD current under ambient light is equal to 0.085 µA, which is negligibly low, so pH analyses can be performed under ambient light. This contrasts with other optical sensors [44], which require the samples being tested to be shielded from ambient light to reduce the signal-to-noise ratio [45].

The sensor can measure pH from 3 to 9, with an error below 0.1 pH unit. Then, at a pH above 9, the PD current stabilizes at about 6 µA. The pH range of 3 to 9 is useful for numerous practical applications. 

The sensing system can be immersed inside any type of liquid container, such as a laboratory beaker (Figure 12), standard or non-standard dimensions of cuvette, etc. An important condition is to obtain the required level of the test specimen, which should be in the range from 4 mm (minimum level) to 8 mm (maximum level), as illustrated in Figure 10, lines 4b and 4a, respectively. Please note that the total level of the test sample in the liquid container is not limited by the maximum sensor immersion level of 8 mm and may exceed this value. The low-level requirement provides conditions when the test specimen fills in the measurement gap, LED and PD fully dipped inside the specimen. The measurement gap is a clearance between the LED and PD which is fixed and equal to 0.8 mm. Meanwhile, the level of the test specimen should not exceed the maximum level of 8 mm. The immersed parts of the PCBs, LED, and PD are electrically insulated from the water up to the maximum level of the liquid. 

In principle, the test specimen can also be poured into a non-standard custom-made cuvette with an inner cavity of 3.6 mm width, 6 mm depth and 4 to 8 mm height, where height is a liquid test specimen level that varies from 4 to 8 mm. In this case, the minimum test specimen volume for a 4 mm sample level is only 55 µL. The minimum volume of the test specimen is a clear advantage for a variety of applications where there is a limited amount of the test material available, or for expensive test materials. Such a customized cuvette can be micromachined from broad varieties of plastic materials such as polycarbonate (PC), cyclic olefin copolymer (COC), polylactic acid (PLA), and polymethyl methacrylate (PMMA) [46]. The selection of a specific material will be based on the required thermo-mechanical material properties and chemical- and/or biocompatibility. The above-mentioned plastics can be processed relatively easily and at a low cost, or micromachined by micromilling or 3D printing, etc. [47].

## 5. Conclusions

We demonstrated a low-cost pH sensor based on the polyaniline (PANI) sensing layer which exhibited sensitivity to the pH variations due to changes in light absorption.

The pH sensor includes a simple optical measuring system. The optical measuring system consists of only an LED as a light source and a PD as a detector, and a sensing layer (a plastic foil covered with PANI). The optical system has good linearity, even at ambient light conditions for which the PD current is negligible. The sensor can perform pH measurements in a liquid specimen in the range of 3 to 9, which is useful for numerous practical applications. Among them are aqueous-based solutions used in chemistry, the food industry [48], agriculture, environmental studies [49], and biomedical applications [48], etc. 

The pH sensor can be used in the field environment, unlike other sensing systems that need strictly controlled laboratory conditions. The sensor requires a tiny volume of the test specimen, as little as 55 µL. The system demonstrates its functionality in transparent solutions, although it must still be tested in non-fully transparent and colored solutions. The pH sensor comprises exclusively commercial off-the-shelf (COTS) components and is assembled using standard surface mounting technology (SMT).

## Data Availability

Data are contained within the article.
